# The Role of Calcium, 25-Hydroxyvitamin D, and Parathyroid Hormone in Irritable Bowel Syndrome: A Bidirectional Two-Sample Mendelian Randomization Study

**DOI:** 10.3390/nu14235109

**Published:** 2022-12-01

**Authors:** Ning Xie, Jiale Xie, Ziwei Wang, Qiuai Shu, Haitao Shi, Jinhai Wang, Na Liu, Feng Xu, Jian Wu

**Affiliations:** 1 Second Affiliated Hospital, Xi’an Jiaotong University, Xi’an 710049, China; 2 Bioinspired Engineering and Biomechanics Center (BEBC), Xi’an Jiaotong University, Xi’an 710049, China; 3 The Key Laboratory of Biomedical Information Engineering of Ministry of Education, School of Life Science and Technology, Xi’an Jiaotong University, Xi’an 710049, China; 4 Department of Joint Surgery, HongHui Hospital, Xi’an Jiaotong University, Xi’an 710054, China; 5 National Clinical Research Center for Digestive Diseases, State Key Laboratory of Cancer Biology, Xijing Hospital of Digestive Diseases, Air Force Medical University, Xi’an 710032, China

**Keywords:** causal effects, irritable bowel syndrome, Mendelian randomization, calcium, vitamin D, parathyroid hormone

## Abstract

Several observational studies have indicated the potential associations among calcium, vitamin D (Vit-D), and irritable bowel syndrome (IBS). However, the causal relationship deduced from these studies is subject to residual confounding factors and reverse causation. Therefore, we aimed to explore the bidirectional causal effects among serum calcium, Vit-D, PTH, and IBS at the genetic level by a two-sample Mendelian randomization (MR) analysis of the datasets from IEU OpenGWAS database. Sensitivity analyses were performed to evaluate the robustness. The estimates were presented as odds ratios (ORs) with their 95% confidence intervals (CIs). The results of the inverse variance weighted method did not reveal any causal relationship between the genetically predisposed calcium (OR = 0.92, 95% CI: 0.80–1.06, *p* = 0.25) and Vit-D (OR = 0.99, 95% CI: 0.83–1.19, *p* = 0.94) level and the risk of IBS. The bidirectional analysis demonstrated that genetic predisposition to IBS was associated with a decreased level of PTH (beta: −0.19, 95%CI: −0.34 to −0.04, *p* = 0.01). In conclusion, the present study indicates no causal relationship between the serum calcium and Vit-D concentrations and the risk of IBS. The potential mechanisms via which IBS affects serum PTH need to be further investigated.

## 1. Introduction

Irritable bowel syndrome (IBS) is one of the most common gastrointestinal diseases, and affects approximately 5–10% of the global population, which exerts an immense impact on the patient’s quality of life, society, and economy [1]. The most complained symptoms include abdominal pain/discomfort and diarrhea/constipation. The pathogenesis of IBS is complex and recent studies bring the consensus that IBS mainly results from the disorder of gut–brain interactions [2]. Furthermore, epidemiological studies suggest that genetics, diet, gut microbiota dysbiosis, gut infection, and psychological factors are all risk factors for IBS, which can exert effects on IBS via disrupting the bidirectional interactions of the gut–brain axis [3,4]. Considering these factors, the common therapeutics for IBS include dietary exclusion, probiotics/fecal microbiota transplant, antibiotics, psychotropic medications, and symptom-relieving drugs (e.g., antispasmodics, antidiarrheal agents, and laxative) [5]. However, all the treatments have limited therapeutic effectiveness. Therefore, there is still an unmet need for improved understanding of the pathophysiological mechanisms of IBS to develop more effective therapeutic approaches.

Recent studies demonstrate that the diet and micronutrients play a vital role in the pathophysiology of IBS, and over 80% of IBS patients report food triggers for their complaints, such as dairy products, gluten, alcohol, and fried foods [6,7]. Noteworthily, dietary fibers are related to the onset of IBS symptoms, as they can exert effects on nutrients bioavailability, gut motility, stool pattern, and the gut microbiota [8]. Specifically, the insoluble fibers, which are poorly absorbed in the gut, can provoke and exacerbate the symptoms of IBS patients, while the soluble fibers can improve stool pattern [9,10]. Furthermore, FODMAPs (fermentable oligosaccharides, disaccharides, monosaccharides, and polyols), which are rich in some vegetables, fruits, dairy products, and legumes, are also associated with the development and severity of symptoms in specific IBS subgroup via their fermentative and osmotic effects on the gut [1,11]. These findings provide some promising dietary therapies including dietary exclusion and dietary supplementation. For example, the increased intake of soluble fibers and reduced intake of insoluble fibers are suggested for IBS subjects [8]. Moreover, a low-FODMAP diet is a recommended therapy for IBS patients by the American College of Gastroenterology. Notably, several epidemiological studies have reported the deficiency of vitamin D (Vit-D) and calcium in IBS patients [7], which indicates that Vit-D and calcium would serve as promising targets for potential dietary therapies.

Calcium homeostasis, which plays a vital role in various cellular and biological processes, is mainly regulated by concerted action of the calciotropic hormones, such as Vit-D and parathyroid hormone (PTH) [12]. Studies indicate that supplementation of Vit-D and calcium might help improve the symptoms of IBS patients [13]. However, randomized controlled trials on the effects of Vit-D and calcium supplementation for IBS patients yielded contradictory results [14,15,16]. Additionally, the causal relationship among Vit-D, calcium, and the risk of IBS needs to be illustrated considering the potential unmeasured confounders or reverse causality in previous observational studies.

Based on Mendel’s law of inheritance, Mendelian randomization (MR) analysis can use genetic variants, namely single-nucleotide polymorphisms (SNPs), as instrumental variables (IVs) to estimate the causal effects of the predefined exposure on outcome [17]. Since genetic variants are randomly allocated at conception and remain stable after birth, MR is less susceptible to confounding factors and reverse causation, thus simulating the randomized controlled trials in the clinic. With the existing genome-wide association study (GWAS) databank, our study is dedicated to probing the causal association between Vit-D, calcium, PTH, and IBS via a bidirectional two-sample MR study.

## 2. Materials and Methods

### 2.1. Study Design

**Figure 1:**
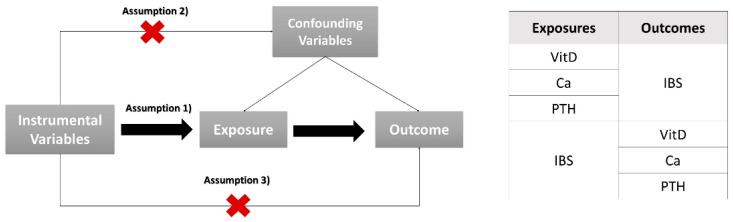
(**Left**): a schematic diagram showing three assumptions of MR; (**Right**): overview of the exposures and outcomes in our MR analysis. VitD, 25-Hydroxyvitamin D; Ca, calcium; PTH, parathyroid hormone; IBS, irritable bowel syndrome.

To investigate the causality between exposures and disease, we conducted two-sample MR analysis that used genetic variants as instrumental variables to explore the causal effects of risk factors on outcomes. Compared with observational studies, MR can avoid reverse causation and reduce confounding factors. The graphical flow of the experimental design is shown in Figure 1.

The validity of MR analysis relies on three assumptions: (1) there is strong association between the IVs and the exposure; (2) each IV is not associated with confounding variables; (3) each IV is only associated with the outcome through the exposure; there are no alternative pathways for the association.

### 2.2. Data Sources and Study Population

The data of our study were obtained from the IEU OpenGWAS database (https://gwas.mrcieu.ac.uk/, accessed on 10 October 2022), a database of 244,879,032,980 genetic associations from 42,334 GWAS summary datasets, for query or download.

The datasets utilized in our study are shown in Table 1. For the dataset of Vit-D, summary statistics of serum 25-hydroxyvitamin D levels were from a GWAS of the EBI database with a sample size of 496,946 (ebi-a-GCST90000618) [18]. For the dataset of calcium, we used summary statistics from a GWAS of UK Biobank from Neale lab with a sample size of 315,153 (ukb-d-30680_irnt). For the dataset of PTH, the complete GWAS summary data on protein levels as described by Sun et al. (2018) was used (prot-a-2431) [19], and the sample size was 3301. For the dataset of IBS, summary statistics from FinnGen biobank analysis including 4605 patients of IBS and 182,423 controls were used (finn-b-K11_IBS) [20]. All cases were defined by the code M13 in the International Classification of Diseases—Tenth Revision (ICD-10).

All the above data samples were of European ethnicity. In all original studies, ethical approval and consent to participate were obtained.

### 2.3. Selection of Instrumental Variables

Firstly, the summary-level data above for Vit-D and calcium were screened by the genome-wide significance (*p* < 5 × 10^−8^) to select the SNPs genetically associated with the traits. To avoid inaccurate results due to too few SNPs, the significance threshold of PTH data was relaxed to 5 × 10^−6^. Secondly, we utilized the linkage disequilibrium clumping to exclude some undesirable SNPs (r^2^ > 0.001). Thirdly, we harmonized the respective exposure and outcome datasets using effect allele frequencies, while removing palindromic SNPs with intermediate allele frequencies. Lastly, according to the third assumption of MR that genetic variation cannot be associated with any possible confounding factor, we used PhenoScanner V2 [21] (a database of human genotype–phenotype associations) to search the SNPs and exclude those associated (*p* < 1 × 10^−5^) with confounding factors such as drinking [22], smoking [23], depression, and anxiety [24].

The IV exposure strength of genetic instruments was assessed from the F statistic using an approximation. If F > 10, there is sufficient strength to avoid a problem of weak instrument bias in the two-sample model. The F statistics were computed by the admittedly reliable formula F = R^2^ (N − 2)/(1 − R^2^). R^2^ and N refer to the cumulative explained variance of selected SNPs and sample size separately [25]. R^2^ was calculated using the formula R^2^ = 2 × MAF × (1 − MAF) × Beta^2^ [26].

### 2.4. Statistical Analyses

Multiple approaches were used in our study. We utilized the method of inverse variance weighted (IVW) as the primary analysis for its efficiency to estimate the causal effect. The weighted median was used as auxiliary method when the heterogeneity was significant, and the MR-Egger regression method was used to assess the pleiotropy by intercept test. According to the assumption of MR analysis, the instrumental variable must be only associated with the outcome through the risk factor; thus, if there are other pathways via which the outcome is influenced by genetic variants, bias will occur, and the horizontal pleiotropy may increase the false positive rate. Therefore, the pleiotropy should be evaluated using the method of MR-Egger and MR-PRESSO. The former can evaluate the potential pleiotropy in the IVW model, and the latter is used for testing horizontal pleiotropy via identifying and removing outlying instrumental variables (NbDistribution = 3000, SignifThreshold = 0.05). The leave-one-out sensitivity analysis was performed to evaluate the robustness of the study findings. The estimates were presented as odds ratios (ORs) with their 95% confidence intervals (CIs) per one standard deviation (SD) increase in the exposures. The statistical analyses above were performed in R 4.1.3 with R package of “TwoSampleMR” (version 0.5.6) and “MRPRESSO”.

## 3. Results

### 3.1. Instrumental Variables

In the analysis investigating the effect of Vit-D and calcium on IBS risk, 110 and 186 SNPs were screened, respectively, as potential instrumental variables (*p* < 5 × 10^−8^). As for PTH, 15 SNPs were screened for instrumental variables (*p* < 5 × 10^−6^). After linkage disequilibrium clumping and the removal of palindromic SNPs and confounders, 102, 176, and 14 SNPs could be used in the analyses as the instrumental variables of Vit-D, calcium, and PTH, respectively. The F statistics demonstrated that there was no bias due to weak instruments (F > 10, Table A1, Table A2, Table A3 and Table A4).

### 3.2. Main Analyses and Sensitivity Analyses

**Figure 2:**
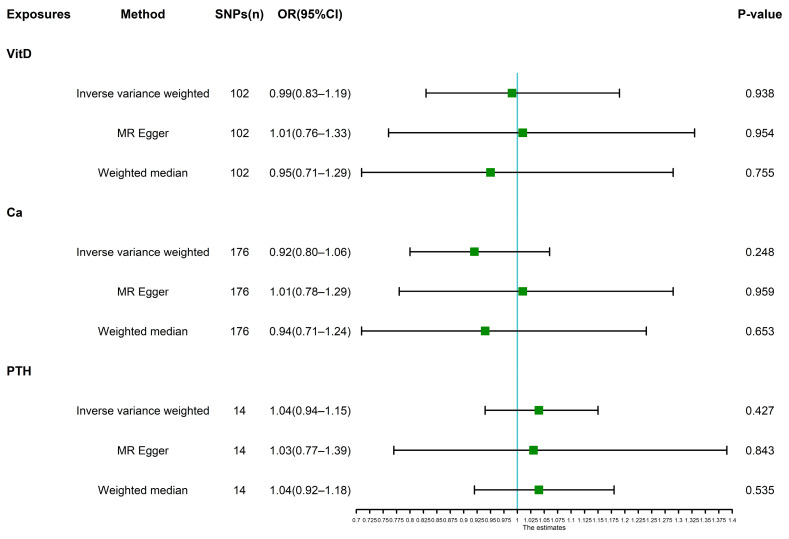
The result of MR analysis investigating the causality between IBS and VitD, Ca, and PTH using multiple approaches. VitD, 25-hydroxyvitamin D; Ca, calcium; PTH, parathyroid hormone; IBS, irritable bowel syndrome; OR, odds ratio; 95% CI, 95% confidence interval.

As shown in Figure 2, genetically predicted risk of IBS was not associated with the levels of vitamin D (*p* = 0.938, OR = 0.99, 95% CI: 0.83–1.19), calcium (*p* = 0.248, OR = 0.92, 95% CI: 0.80–1.06), and parathyroid hormone (*p* = 0.427, OR = 1.04, 95% CI: 0.94–1.15) using the IVW method. As shown in Table 2, Cochran’s Q statistics demonstrated no heterogeneity based on genetically predicted SNPs of Vit-D, calcium, and PTH (*p >* 0.05). The MR Egger intercept test showed no evidence of directional pleiotropy (*p >* 0.05). The results of the leave-one-out method demonstrated that the removal of SNP did not fundamentally affect the results, which indicated that the results were actually robust.

**Figure 3:**
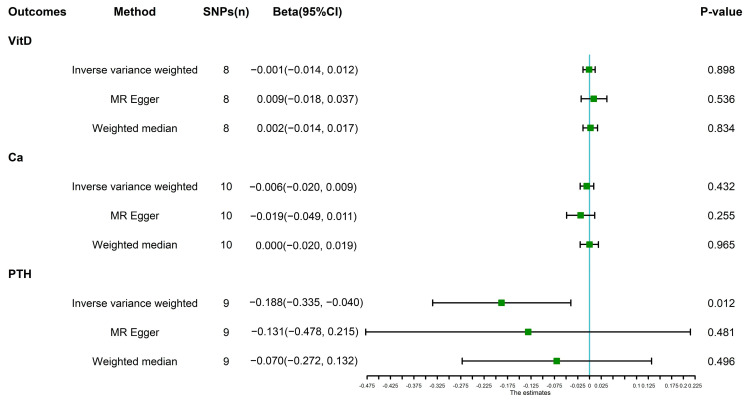
The result of MR analysis investigating the causality between VitD, Ca, and PTH and IBS using multiple approaches. VitD, 25-hydroxyvitamin D; Ca, calcium; PTH, parathyroid hormone; IBS, irritable bowel syndrome; 95% CI, 95% confidence interval.

As shown in Figure 3, genetically predicted levels of PTH were associated with the risk of IBS (*p* = 0.012, Beta = −0.188) while IBS was not the risk factor of Vit-D (*p* = 0.898, Beta = −0.001) and calcium (*p* = 0.432, Beta = −0.006) using the IVW method. As shown in Table 2, Cochran’s Q statistics demonstrated no heterogeneity based on genetically predicted SNPs of Vit-D, calcium, and PTH (*p >* 0.05). The MR Egger intercept test showed no evidence of directional pleiotropy (*p >* 0.05). The results of the leave-one-out method demonstrated that the removal of SNP did not fundamentally affect the results, which indicated that the results were actually robust.

## 4. Discussion

To the best of our knowledge, this is the first two-sample MR study to generally clarify the causal relationship among calcium, Vit-D, PTH, and IBS. Despite employing the latest large sample size and strong instruments, our MR results did not indicate the significantly causal associations of genetically predicted calcium, Vit-D, and PTH with the risk of IBS.

Researchers have been devoted to exploring the role of micronutrients in the pathogenesis and treatment of IBS [27,28]. A systematic review including 12 interventional and 14 observational studies showed that IBS patients generally had lower levels of Vit-D, vitamin B2, calcium, and iron compared with control subjects. Meanwhile, studies also found that exclusion diets were associated with deficiencies of the aforementioned micronutrients [7]. As the major circulating form of Vit-D, 25-hydroxyvitamin D is used as indicator of Vit-D status. 25-Hydroxyvitamin D is critical to regulate calcium metabolism and a series of pathological and physiological processes in intestinal homeostasis [29]. The various effects of Vit-D supplementation on IBS patients were reported in several randomized controlled trials and systematic reviews. Jalili et al. conducted a randomized, double-blind, placebo-controlled clinical trial to assess the impact of Vit-D supplementation on symptoms severity and quality of life (QOL) in IBS patients and found that, compared to the placebo group, Vit-D therapy could markedly improve the symptoms and QOL of IBS patients [14]. Similarly, a systematic review and meta-analysis including four randomized, placebo-controlled trials showed that Vit-D supplementation was remarkably superior to placebo in improving the symptom severity (WMD: −84.21, 95% CI: −111.38 to −57.05, I^2^ = 73.2%; WMD: −28.29, 95% CI: −49.95 to −6.62, I^2^ = 46.6%, respectively) and QOL (WMD: 14.98, 95% CI: 12.06 to 17.90, I^2^ = 0.0%; WMD: 6.55, 95% CI: −2.23 to 15.33, I^2^ = 82.7%, respectively) of IBS patients [30]. However, the other randomized, double-blind, placebo-controlled study by Williams et al. demonstrated that there were no improvements in the IBS symptom severity and QOL between the trial (Vit-D supplementation) and placebo groups [15]. Moreover, a systematic review and meta-analysis based on six randomized controlled trials including 616 participants indicated that Vit-D supplementation led to no significant improvements in symptom severity and QOL of IBS subjects in contrast to placebo [31]. Considering that Vit-D contributes to the regulation of the gut microbiome, immune system, inflammatory processes, and the intestinal mucosal barrier, the present interventional trials on IBS mainly focused on Vit-D supplementation. Few studies evaluated the effects of calcium supplementation on IBS symptom severity and QOL. In contrast to studies that reported the relationship among Vit-D, calcium, and IBS, our study suggested no causal association among Vit-D, calcium, and IBS. The contradictory findings might be explained by several factors: trial participants with different ages, races, sexes, and vitamin D statuses, sample size, intervention duration, intervention diet, and placebo effects.

In addition, our bidirectional two-sample MR analysis identified that IBS was associated with a lower level of PTH, although there was no causal effect of PTH on IBS. The main function of PTH is to increase the concentration of serum calcium and decrease the concentration of serum phosphorus by impacting its primary target organs of bone and kidney, so as to regulate the homeostasis of calcium and phosphorus in vivo. It was noteworthy that recent studies suggested an increased risk of osteoporosis and osteoporotic fracture for IBS patients. A systematic review and meta-analysis including four cohorts and one cross-sectional study with 526,633 participants indicated that IBS patients had a remarkably higher risk of osteoporosis than the non-IBS subjects (pooled RR: 1.95, 95%CI: 1.04–3.64, I^2^ = 100%) [32]. Moreover, even though not statistically significant, IBS subjects had an increased risk of osteoporotic fracture (pooled RR: 1.58, 95%CI: 0.95–2.62, I^2^ = 99%). The possible mechanisms for the association between IBS and osteoporosis comprise chronic inflammation, abnormal activation of the hypothalamic–pituitary–adrenal (HPA) axis, smoking, and malnutrition. When suffering from osteoporosis, the secretion of PTH was reduced to inhibit the activity of osteoclasts, thus impeding the progression of osteoporosis, which might be the plausible explanation for the relationship between IBS and reduced level of PTH.

To our knowledge, this is the first study to elucidate the causal correlation among calcium, Vit-D, PTH, and IBS from the perspective of genetic variants using a bidirectional two-sample MR approach. This method could greatly circumvent the possible impacts of reverse causation and residual confounding factors, such as incomplete adjustment for confounders, the absence of high-quality evidence, and relatively small sample sizes of trials. Additionally, we performed several sensitivity analyses to strengthen the robustness of our results.

However, there are some limitations associated with this study. Firstly, the study mainly analyzed the European participants enrolled in the GWAS biobank; hence, the results could not precisely reflect the fact of patients from other regions and races. Secondly, we failed to accomplish the sex-specific, IBS subtype-specific, age-specific, and race-specific analyses due to a lack of data. Lastly, MR analysis possesses some inherent shortcomings, making it impossible to eliminate the effects of confounding factors and horizontal pleiotropy.

In conclusion, the present study provides no evidence that calcium, Vit-D, and PTH are causally associated with IBS, and it suggests a lower concentration of PTH in IBS subjects. Our findings may reduce possible expenses and research interests in elucidating the effects of calcium, Vit-D, and PTH on IBS. More importantly, further research is needed to investigate the causal relationship between micronutrients and IBS.

## Figures and Tables

**Table 1:** The information of datasets used in our study.

Traits	GWAS ID	Author	PMID	Ancestor	Sample Size
VitD	ebi-a-GCST90000618	Revez et al.	32242144	European	496,946
Ca	ukb-d-30680_irnt	Neale lab	NA	European	315,153
PTH	prot-a-2431	Sun et al.	29875488	European	3301
IBS	finn-b-K11_IBS	NA	NA	European	187,028

Abbreviations: VitD, 25-hydroxyvitamin D; Ca, calcium; PTH, parathyroid hormone; IBS, irritable bowel syndrome.

**Table 2:** The result of sensitivity analyses of MR.

Exposure-Outcome	MR-PRESSO	IVW Estimates		MR-Egger Pleiotropy Test	
	Global *p*-Value	Cochran’s Q	*p*-Value	MR-Egger Intercept	*p*-Value
VitD-IBS	0.384	102.68	0.435	−0.001	0.886
Ca-IBS	0.670	166.66	0.662	−0.003	0.417
PTH-IBS	0.555	12.79	0.464	0.002	0.949
IBS-VitD	0.645	5.329	0.620	−0.002	0.452
IBS-Ca	0.617	6.515	0.687	0.002	0.361
IBS-PTH	0.480	8.100	0.424	−0.009	0.732

Abbreviations: VitD, 25-hydroxyvitamin D; Ca, calcium; PTH, parathyroid hormone; IBS, irritable bowel syndrome.

## Data Availability

This study is based on the public database, and all related-datasets are available at https://gwas.mrcieu.ac.uk/ (accessed on 10 October 2022).

## Appendix A

**Table A1:** The F statistics for SNPs strongly associated with vitamin D.

SNP	Eaf	Beta	R^2^	F
rs10277163	0.255	−0.014	0.0001	39
rs1038165	0.579	0.012	0.0001	32
rs1042034	0.792	−0.015	0.0001	37
rs10438978	0.820	−0.017	0.0001	43
rs1047891	0.317	−0.013	0.0001	39
rs1048328	0.080	0.031	0.0001	72
rs10859995	0.580	−0.044	0.0009	461
rs11023159	0.033	0.048	0.0001	73
rs11076175	0.176	0.023	0.0002	76
rs111515741	0.017	−0.049	0.0001	40
rs11207969	0.351	0.021	0.0002	99
rs11264361	0.251	0.017	0.0001	57
rs11542462	0.134	−0.025	0.0001	71
rs11600054	0.010	0.068	0.0001	46
rs11726886	0.291	−0.054	0.0012	591
rs117300835	0.013	−0.335	0.0029	1469
rs11791258	0.191	0.014	0.0001	30
rs11867297	0.385	0.014	0.0001	43
rs12056768	0.584	−0.023	0.0003	130
rs12283049	0.234	−0.056	0.0011	569
rs12324720	0.175	−0.015	0.0001	32
rs12462826	0.370	−0.013	0.0001	40
rs12501515	0.590	−0.079	0.0030	1504
rs12775091	0.213	0.016	0.0001	40
rs1321247	0.102	−0.022	0.0001	45
rs13294734	0.466	0.013	0.0001	39
rs138335	0.659	−0.014	0.0001	42
rs1384687	0.132	−0.017	0.0001	32
rs142004400	0.034	−0.031	0.0001	32
rs142158911	0.112	0.026	0.0001	68
rs1532085	0.617	0.025	0.0003	150
rs1627043	0.033	−0.049	0.0002	76
rs1684600	0.299	−0.013	0.0001	33
rs17207784	0.324	−0.013	0.0001	40
rs17473257	0.017	−0.061	0.0001	63
rs1800588	0.215	−0.031	0.0003	156
rs1858889	0.503	0.013	0.0001	45
rs1871395	0.153	−0.020	0.0001	53
rs1949633	0.606	0.011	0.0001	31
rs2037511	0.166	0.018	0.0001	43
rs2074735	0.065	0.029	0.0001	52
rs2229742	0.105	−0.025	0.0001	58
rs2245133	0.164	−0.021	0.0001	62
rs2297991	0.718	0.013	0.0001	33
rs2494429	0.823	−0.015	0.0001	32
rs2511279	0.960	0.098	0.0007	365
rs2595644	0.385	−0.012	0.0001	35
rs2710651	0.526	−0.012	0.0001	33
rs2807834	0.685	−0.015	0.0001	49
rs28435470	0.663	−0.012	0.0001	31
rs2847500	0.123	−0.023	0.0001	55
rs325393	0.278	−0.014	0.0001	37
rs34186890	0.260	−0.016	0.0001	47
rs34726834	0.252	0.014	0.0001	37
rs35270497	0.176	0.016	0.0001	35
rs3732220	0.085	−0.048	0.0004	177
rs3829251	0.133	−0.114	0.0030	1508
rs4147536	0.789	−0.015	0.0001	36
rs4348160	0.327	−0.026	0.0003	146
rs4364259	0.199	0.017	0.0001	47
rs4420638	0.177	−0.019	0.0001	54
rs4580037	0.286	−0.014	0.0001	37
rs512083	0.462	0.012	0.0001	37
rs5770794	0.314	−0.013	0.0001	38
rs6129648	0.380	0.014	0.0001	46
rs61698755	0.560	−0.011	0.0001	32
rs61747728	0.039	0.030	0.0001	34
rs61813875	0.025	0.082	0.0003	162
rs61887421	0.030	−0.037	0.0001	39
rs62007299	0.713	−0.012	0.0001	31
rs62129966	0.161	0.061	0.0010	502
rs635634	0.187	−0.015	0.0001	34
rs6438900	0.256	0.015	0.0001	43
rs6672758	0.800	0.016	0.0001	42
rs6834488	0.423	−0.014	0.0001	51
rs71599974	0.148	0.026	0.0002	83
rs727857	0.612	−0.012	0.0001	34
rs733454	0.099	0.019	0.0001	32
rs73413596	0.074	0.022	0.0001	34
rs742493	0.113	0.018	0.0001	34
rs7528419	0.224	0.022	0.0002	80
rs7569755	0.289	0.014	0.0001	38
rs7580771	0.176	−0.017	0.0001	40
rs7712001	0.440	0.012	0.0001	35
rs77532868	0.052	0.026	0.0001	33
rs77924615	0.194	−0.015	0.0001	36
rs77960347	0.013	−0.053	0.0001	34
rs78649910	0.106	−0.019	0.0001	34
rs8018720	0.824	−0.034	0.0003	172
rs8107974	0.076	0.036	0.0002	89
rs8121940	0.198	−0.044	0.0006	299
rs9375037	0.443	0.012	0.0001	34
rs9409266	0.862	−0.017	0.0001	33
rs964184	0.867	0.041	0.0004	189
rs9847248	0.713	−0.012	0.0001	31
rs986649	0.322	0.013	0.0001	36
rs9946771	0.066	−0.023	0.0001	34

**Table A2:** The F statistics for SNPs strongly associated with calcium.

SNP	Eaf	Beta	R^2^	F
rs10108887	0.388	0.014	0.0001	31
rs1036332	0.739	0.022	0.0002	59
rs1061134	0.092	−0.025	0.0001	33
rs10754439	0.417	0.014	0.0001	32
rs10819178	0.638	0.032	0.0005	153
rs10858935	0.687	−0.019	0.0002	51
rs10917386	0.690	0.020	0.0002	54
rs10958700	0.247	0.020	0.0002	48
rs11078597	0.186	0.051	0.0008	249
rs11085015	0.800	−0.018	0.0001	33
rs112174050	0.025	0.102	0.0005	163
rs114949263	0.112	0.034	0.0002	72
rs11588907	0.342	−0.016	0.0001	36
rs11616030	0.087	−0.031	0.0002	48
rs11621792	0.454	0.016	0.0001	39
rs11629876	0.331	−0.018	0.0001	46
rs11671393	0.040	−0.045	0.0002	48
rs116769926	0.024	0.073	0.0002	76
rs117080418	0.011	−0.099	0.0002	64
rs117179023	0.012	−0.068	0.0001	34
rs117213754	0.015	0.104	0.0003	100
rs11730491	0.168	0.021	0.0001	38
rs11743466	0.364	0.018	0.0001	45
rs117896857	0.027	−0.055	0.0002	51
rs11792928	0.295	−0.017	0.0001	36
rs12132412	0.388	0.028	0.0004	121
rs12135382	0.584	0.022	0.0002	76
rs12147703	0.895	−0.028	0.0001	47
rs12339541	0.064	−0.061	0.0004	141
rs12378991	0.080	−0.038	0.0002	67
rs12583851	0.751	−0.023	0.0002	60
rs12613807	0.444	0.016	0.0001	40
rs12675477	0.274	0.017	0.0001	37
rs12793731	0.511	0.015	0.0001	34
rs12918968	0.439	−0.032	0.0005	157
rs12922549	0.237	−0.022	0.0002	55
rs12933858	0.493	0.019	0.0002	56
rs12982234	0.040	−0.058	0.0003	82
rs12998379	0.194	−0.024	0.0002	58
rs13073106	0.641	0.038	0.0007	205
rs13389219	0.394	−0.016	0.0001	40
rs1354034	0.601	−0.018	0.0002	49
rs1374161	0.491	−0.023	0.0003	80
rs138789759	0.075	0.047	0.0003	96
rs147233090	0.024	0.103	0.0005	157
rs1476698	0.369	−0.021	0.0002	62
rs1497826	0.373	0.024	0.0003	86
rs149807892	0.016	0.066	0.0001	43
rs1500187	0.456	−0.014	0.0001	29
rs164751	0.410	−0.019	0.0002	54
rs165316	0.198	−0.018	0.0001	31
rs1672991	0.934	0.075	0.0007	221
rs17132144	0.093	−0.026	0.0001	35
rs17164683	0.270	−0.021	0.0002	55
rs17580	0.049	0.047	0.0002	64
rs1763519	0.607	−0.032	0.0005	149
rs17774672	0.158	−0.027	0.0002	62
rs17884869	0.025	−0.111	0.0006	190
rs1801282	0.120	−0.037	0.0003	92
rs1858800	0.345	0.031	0.0004	134
rs2001884	0.507	−0.017	0.0001	46
rs2004315	0.625	0.033	0.0005	160
rs2241699	0.277	−0.026	0.0003	84
rs2243010	0.206	−0.018	0.0001	32
rs2249825	0.269	−0.016	0.0001	33
rs2274224	0.432	−0.017	0.0001	47
rs2309233	0.731	0.021	0.0002	56
rs2327774	0.378	−0.022	0.0002	71
rs2335534	0.179	−0.038	0.0004	136
rs2343592	0.267	−0.023	0.0002	67
rs2370218	0.766	−0.022	0.0002	53
rs2419886	0.257	−0.020	0.0001	47
rs255755	0.270	0.015	0.0001	29
rs2647242	0.798	−0.020	0.0001	40
rs2762938	0.587	0.016	0.0001	37
rs28520334	0.119	0.022	0.0001	31
rs28929474	0.020	0.123	0.0006	189
rs2971855	0.302	0.019	0.0002	49
rs3011642	0.243	0.020	0.0001	45
rs3026445	0.367	−0.018	0.0001	45
rs302650	0.432	−0.019	0.0002	58
rs3091842	0.044	0.094	0.0007	235
rs3133548	0.142	0.020	0.0001	31
rs34042070	0.186	0.021	0.0001	42
rs34066945	0.358	−0.026	0.0003	97
rs34290411	0.286	0.018	0.0001	41
rs34395935	0.152	0.059	0.0009	285
rs35118755	0.149	0.023	0.0001	43
rs35590487	0.241	−0.021	0.0002	53
rs35751693	0.039	0.043	0.0001	45
rs35852840	0.059	0.029	0.0001	29
rs36086195	0.580	0.020	0.0002	63
rs36104352	0.121	0.028	0.0002	52
rs3748861	0.202	−0.017	0.0001	31
rs3795243	0.126	0.022	0.0001	32
rs3822858	0.407	−0.016	0.0001	40
rs3931841	0.681	−0.026	0.0003	94
rs4082330	0.814	0.021	0.0001	41
rs41278174	0.027	0.050	0.0001	42
rs41393948	0.113	−0.022	0.0001	29
rs4239142	0.745	−0.017	0.0001	35
rs4320103	0.039	0.045	0.0002	49
rs4324076	0.530	−0.018	0.0002	53
rs4633480	0.556	0.020	0.0002	62
rs4721467	0.738	−0.021	0.0002	54
rs4744854	0.629	−0.033	0.0005	156
rs4758621	0.308	−0.026	0.0003	94
rs4790310	0.575	−0.020	0.0002	59
rs4841132	0.908	0.057	0.0005	171
rs4938642	0.074	0.034	0.0002	49
rs4976647	0.332	0.018	0.0001	47
rs498490	0.164	−0.031	0.0003	82
rs55772024	0.242	−0.017	0.0001	32
rs56406311	0.386	0.018	0.0002	47
rs567743	0.708	0.015	0.0001	29
rs5751350	0.330	0.016	0.0001	37
rs5760495	0.354	0.017	0.0001	41
rs5786388	0.581	0.022	0.0002	71
rs58579887	0.404	0.016	0.0001	40
rs59821684	0.029	0.054	0.0002	52
rs6127099	0.278	−0.053	0.0011	358
rs62134679	0.148	0.022	0.0001	39
rs62211622	0.184	−0.018	0.0001	32
rs62309863	0.586	−0.014	0.0001	32
rs62472728	0.060	0.032	0.0001	36
rs634916	0.511	−0.014	0.0001	31
rs648514	0.467	−0.014	0.0001	30
rs6580981	0.458	−0.018	0.0002	52
rs66920316	0.196	−0.021	0.0001	45
rs6741561	0.393	−0.038	0.0007	222
rs6771438	0.116	−0.027	0.0002	48
rs6841429	0.166	−0.040	0.0004	137
rs6909201	0.517	−0.042	0.0009	281
rs710217	0.485	0.024	0.0003	94
rs71565393	0.177	0.018	0.0001	30
rs7221118	0.215	−0.020	0.0001	41
rs722298	0.438	0.015	0.0001	34
rs72660383	0.064	−0.028	0.0001	30
rs72697816	0.163	0.020	0.0001	33
rs73001065	0.071	0.036	0.0002	53
rs73186030	0.128	0.193	0.0083	2637
rs73186098	0.015	−0.091	0.0002	78
rs73536752	0.043	−0.033	0.0001	29
rs7370877	0.420	−0.017	0.0001	44
rs7402977	0.267	−0.016	0.0001	30
rs74230087	0.078	−0.055	0.0004	134
rs7533348	0.352	−0.021	0.0002	61
rs7546838	0.651	0.020	0.0002	55
rs7559013	0.130	0.025	0.0001	46
rs75702986	0.188	0.037	0.0004	134
rs7587636	0.529	−0.016	0.0001	38
rs75895430	0.033	0.080	0.0004	126
rs7592216	0.869	−0.020	0.0001	30
rs7599	0.634	−0.019	0.0002	55
rs7688574	0.369	0.015	0.0001	32
rs7730344	0.293	0.017	0.0001	40
rs77542162	0.023	−0.089	0.0004	113
rs777588	0.578	−0.029	0.0004	126
rs778368	0.585	−0.014	0.0001	30
rs7786368	0.416	−0.024	0.0003	90
rs7864156	0.390	0.019	0.0002	55
rs7913072	0.859	−0.021	0.0001	33
rs7924737	0.349	−0.015	0.0001	34
rs7940215	0.434	0.014	0.0001	30
rs79501693	0.021	−0.050	0.0001	32
rs8041057	0.714	−0.015	0.0001	30
rs835664	0.456	−0.017	0.0001	46
rs838717	0.566	−0.046	0.0010	327
rs883951	0.261	0.023	0.0002	66
rs928760	0.303	−0.018	0.0001	45
rs9388399	0.312	−0.024	0.0003	81
rs9399697	0.464	0.014	0.0001	32
rs9419741	0.479	0.015	0.0001	36
rs945890	0.714	−0.017	0.0001	37
rs949300	0.380	0.015	0.0001	35
rs9530	0.549	−0.031	0.0005	149
rs9532958	0.855	0.035	0.0003	99
rs9611396	0.655	−0.014	0.0001	29
rs9895661	0.831	−0.028	0.0002	69

**Table A3:** The F statistics for SNPs strongly associated with parathyroid hormone.

SNP	Eaf	Beta	R^2^	F
rs10886704	0.425	0.121	0.007	24
rs10902764	0.576	0.131	0.008	28
rs150350229	0.026	−0.388	0.008	25
rs16895559	0.043	0.286	0.007	22
rs17267730	0.045	0.292	0.007	24
rs2585442	0.249	−0.158	0.009	31
rs34421071	0.171	−0.155	0.007	23
rs464202	0.888	−0.181	0.007	22
rs62533188	0.106	−0.191	0.007	23
rs678360	0.962	−0.346	0.009	29
rs73556612	0.035	0.328	0.007	24
rs7514637	0.788	−0.148	0.007	24
rs753409	0.201	0.151	0.007	24
rs9316680	0.384	−0.137	0.009	30
rs950455	0.317	0.129	0.007	24

**Table A4:** The F statistics for SNPs strongly associated with irritable bowel syndrome.

SNP	Eaf	Beta	R^2^	F
rs10275986	0.316	−0.113	0.005	1028
rs10985554	0.394	0.103	0.005	947
rs11952072	0.113	0.157	0.005	932
rs12570677	0.112	−0.158	0.005	935
rs12956689	0.400	−0.102	0.005	930
rs147367149	0.010	−0.521	0.006	1042
rs177503	0.358	−0.105	0.005	951
rs45506200	0.015	0.416	0.005	957
rs735820	0.001	2.070	0.009	1623
rs763614	0.329	0.107	0.005	948
